# The Expression Pattern and Clinical Significance of the Immune Checkpoint Regulator VISTA in Human Breast Cancer

**DOI:** 10.3389/fimmu.2020.563044

**Published:** 2020-10-29

**Authors:** Xiaoxue Xie, Junying Zhang, Zhongyuan Shi, Wanmei Liu, Xinlei Hu, Chenxin Qie, Wenting Chen, Yan Wang, Li Wang, Jingwei Jiang, Jun Liu

**Affiliations:** ^1^ Jiangsu Key Lab of Drug Screening, China Pharmaceutical University, Nanjing, China; ^2^ Clinical Cancer Research Center, Jiangsu Cancer Hospital and Jiangsu Institute of Cancer Research and The Affiliated Cancer Hospital of Nanjing Medical University, Nanjing, China; ^3^ Department of Pathology, Jiangsu Province Hospital on Integration of Chinese and Western Medicine, Nanjing, China; ^4^ Department of Pathology, The Second Affiliated Hospital of Nanjing Medical University, Nanjing, China; ^5^ Department of Translational Hematology and Oncology Research (THOR), Cleveland Clinic Foundation, Cleveland, OH, United States

**Keywords:** V domain immunoglobulin suppressor of T cell activation, tumor immune microenvironment, single cell RNA-seq, breast cancer, quantitative immunofluorescence

## Abstract

**Background:**

Immunotherapies targeting CTLA-4 and PD-1 have elicited promising responses in a variety of cancers. However, the relatively low response rates warrant the identification of additional immunosuppressive pathways. V domain immunoglobulin suppressor of T cell activation (VISTA) plays a critical role in antitumor immunity and is a valuable target in cancer immunotherapy.

**Methods:**

Here, we used single-cell RNA-seq to analyze the gene expression levels of 14897 cells from a breast cancer sample and its paired 7,320 normal cells. Then, we validated the protein expression of immune checkpoint molecules (VISTA, PD-1, PD-L1, TIGIT, TIM3, and LAG3) in 324 human breast cancer samples by immunohistochemistry and quantitative immunofluorescence (QIF) approaches.

**Results:**

Single cell RNA-seq results show a higher level of immune checkpoint VISTA expression in breast cancer tissue compared to adjacent normal tissue. We also found that VISTA expressed highest in breast cancer tissue than other immune-checkpoints. Immunohistochemical results showed that VISTA was detected with a membranous/cytoplasmic staining pattern in intratumoral immune cells and breast cancer cells. Additionally, VISTA was positively correlated with pathological grade, lymph node status and the levels of PD-1 according to the chi-square test or Fisher’s test. Furthermore, VISTA expression was higher in CD68+ tumor-associated macrophages (TAMs) than in CD4+ T cells, CD8+ cytotoxic T cells or CD20+ B cells.

**Conclusions:**

These findings therefore support the immunoregulatory role of VISTA in breast cancer and indicate that targeting VISTA may benefit breast cancer immunotherapy.

## Background

Globally, breast cancer is one of the most commonly diagnosed cancer types among women, and it accounts for 17.07% of all cancer types among the Chinese population (1). Current mainstream treatments for breast cancer include surgery, radiation therapy, chemotherapy, hormone therapy and targeted therapy (2). These therapies are not efficacious in all types of breast cancer due to a variety of resistance mechanisms and toxicity. On the other hand, immunotherapy for refractory breast cancer, especially for late-stage breast cancer and triple-negative breast cancer (TNBC) has become a promising strategy.

Immune checkpoint receptors transmit co-inhibitory signaling to inhibit T cell activation, thereby controlling the duration and intensity of the immune response. Multiple immune checkpoint proteins, such as PD-1/PD-L1, CTLA-4, TIM3, VISTA, TIGIT, LAG3, and BTLA, have been discovered (3–5). Drugs targeting immune checkpoint molecules, including nivolumab, ipilimumab, and pembrolizumab, have been approved by the U.S. Food and Drug Administration (FDA) and have become breakthrough therapies for cancer (6–8). However, the relatively low response rates (less than 30%) of current immunotherapeutic drugs remain a critical challenge that warrants efforts to identify and overcome additional immunosuppressive pathways (9). V-domain immunoglobulin (Ig) suppressor of T cell activation (VISTA) is a B7-family immune checkpoint protein that plays a multifaceted role in regulating peripheral tolerance, autoimmunity, inflammation, and antitumor immunity (10). Although the extracellular domain of VISTA is homologous to that of PD-L1 (11, 12), our previous study indicates that the VISTA and PD-1/PD-L1 checkpoint pathways are functionally distinct and that they non-redundantly regulate T cell function and the antitumor immune response (12). VISTA is highly expressed on tumor-infiltrating myeloid cells (i.e., CD11b^+^ cells, macrophages, and myeloid-derived suppressor cells (MDSCs)) and tumor-infiltrating T cells (13–16). Our previous study showed high VISTA expression on tumor-infiltrating neutrophils in human pancreatic cancer (17). Jorge Blando et al. reported that VISTA was preferentially expressed at a relatively high level in pancreatic cancer (14). Other studies have revealed that VISTA expression is detected in gastric carcinoma, oral squamous carcinoma, non-small cell lung cancer, ovarian cancer and colorectal cancer (18–22). Among melanoma and prostate cancer patients treated with ipilimumab, VISTA expression is significantly upregulated on CD4^+^ T cells, CD8^+^ T cells and CD68^+^ macrophages, indicating that VISTA may contribute to mechanisms of resistance to checkpoint inhibitor therapies in these cancers (23, 24).

In breast cancer, significant heterogeneity in the immune cell composition is observed across tumor subtypes and patients. The anti-PD-L1 antibody drug TECENTRIQ (atezolizumab) was approved by the FDA in combination with the chemotherapeutic drug Abraxane for the treatment of locally advanced or metastatic TNBC recently. This is the first immunotherapeutic drug approved for breast cancer treatment (25). In this study, we employ single-cell RNA-seq (scRNA-seq) analysis to explore immune checkpoint VISTA, PD-1, PD-L1, TIGIT, TIM3, LAG3 expression in immune cell subsets in human breast tumors. Our analyses revealed significantly increased expression of VISTA in tumor cells compared to normal breast tissue. We also analyzed the expression characters of other immune checkpoints such as PD-1, PD-L1, TIM3, LAG3, and TIGIT in breast cancer tissue. Furthermore, combined with immunohistochemistry and quantitative immunofluorescence, we define the protein expression of VISTA and its relationship with other immune-checkpoints in the breast cancer environment. Our study assessed the expression of VISTA and other immune checkpoint molecules in human breast cancer, and also examined whether the expression levels of VISTA were prognostic in breast cancer patients.

## Methods

### Tissue Sample Source

The human breast cancer obtained from JIANGSU CANCER HOSPITAL for sc-RNA seq was collected from a woman undergoing surgery for primary breast cancer. Adjacent normal tissue was obtained from contralateral prophylactic mastectomies of the same cancer patient. The human breast cancer tissue microarrays used in this experiment were obtained from Shanghai Outdo Biotech Co., Ltd. The tissue samples were fixed in a formalin solution and embedded in paraffin to prepare 4-μm sections of breast cancer tissue for immunohistochemical (IHC) analysis. There were 4 samples of normal breast tissue, 2 samples of breast adenosis tissue, 13 samples of paracancerous tissue and 324 samples of breast cancer tissue. Clinical features, including age, pathological grade, clinical stage, T-category, M-category, and lymph node status, were recorded for each patient.

### Ethics Statement

The study was conducted in accordance with the Declaration of Helsinki, and the protocol was approved by the Medical Ethics Committee of the Shanghai Outdo Biotech Company and performed according to institutional guidelines (No. YB M-05-02).

### Single-Cell Sequencing

See Online methods. The cDNA/DNA/Small RNA libraries were sequenced on the Illumina sequencing platform by Guangzhou Kidio Biotechnology Co., Ltd. (Guangzhou, China). The raw reads were deposited into the NCBI Sequence Read Archive database (Accession Number: SPR234770). Other data and analytical methods are available from the corresponding authors upon reasonable request (**Additional file 1: Supplemental materials and methods**).

### Immunohistochemistry (IHC) Staining

The purchased human breast cancer tissue microarrays were dewaxed in an oven at 65°C for 1 h and then immersed in different concentrations of xylene for rehydration. The sections were subjected to high-temperature antigen retrieval in a 10-mM citrate restorative solution (pH = 6.0). After cooling, the samples were immersed in 3% hydrogen peroxide for 10 min to quench endogenous peroxidase activity. The blocking of nonspecific binding was performed with ready-to-use normal goat serum (AR0009; BOSTER Biological Technology Co.,Ltd.) for 1 h at room temperature. The sections were then incubated with an anti-human VISTA antibody (1:200 dilution) (#64953; Cell Signaling Technology), anti-CD8 antibody (1:100 dilution) (Abcam, ab4055), anti-PD-1 antibody [NAT 105] (1:50 dilution) (Abcam, ab52587), or anti-PD-L1 antibody [28-8] (1:200 dilution) (Abcam, ab205921) overnight at 4°C. Subsequently, a biotinylated immunoglobulin G secondary antibody solution and avidin-biotin peroxidase reagent (Elivision™ Super HRP (Mouse/Rabbit) IHC Kit, KIT-9921, Maixin-Bio) were added to the slides. The chromogenic reaction was visualized by incubation with 3,30-diaminobenzidine (Signal Stain^®^ DAB Substrate Kit #8059; Cell Signaling Technology) for 0-3 min. Hematoxylin was then used as the nuclear counterstain. After dehydration, the sections were mounted in neutral resin and covered with coverslips. In this experiment, human tonsil tissue was used as a positive control experiment for antibody immunohistochemistry (results not shown).

### Multiplex Staining and Multispectral Imaging to Identify the Cell Subsets Expressing VISTA in the Breast Cancer TME

To identify the cell subsets expressing VISTA in the TME, multiplex immunofluorescence staining was obtained using TSA Plus Fluorescence Kits (Panovue, Beijing, China) combined with immunohistochemistry (TSA-IHC). Different primary antibodies were sequentially applied, followed by horseradish peroxidase-conjugated secondary antibody incubation and tyramine signal amplification. The slides were microwave heat treated after each TSA operation. Nuclei were stained with 4′-6′-diamidino-2-phenylindole (DAPI; Sigma-Aldrich) after all the human antigens had been labeled.

### Multiplexed Quantitative Immunofluorescence to Identify the Expression Differences Between VISTA and Other Immune Checkpoint Molecules (TIGIT, TIM3, and LAG3) in Breast Cancer

To identify the expression differences between VISTA and other immune checkpoint molecules (TIGIT, TIM3, and LAG3), multiplex immunofluorescence staining was performed using TSA Plus Fluorescence Kits (Panovue, Beijing, China) combined with immunohistochemistry (TSA-IHC). Different primary antibodies (an anti-human VISTA antibody (#64953; Cell Signaling Technology); an anti-TIGIT antibody [BLR0475] (Abcam, ab243903); an anti-TIM3 antibody (#45203; Cell Signaling Technology); and a rabbit monoclonal antibody [EPR20261] against LAG3 (Abcam, ab209236)) were sequentially applied, followed by horseradish peroxidase-conjugated secondary antibody incubation and tyramide signal amplification. The slides were microwave heat treated after each TSA operation. Nuclei were stained with DAPI (Sigma-Aldrich) after all the human antigens had been labeled.

### Evaluation of Immunostaining

The tissue microarrays from the immunohistochemistry experiments were converted into high-resolution digital sections by a digital pathological section scanner (Nanozoomer XR; Hamamatsu Photonics, Japan). Five high-powered fields (400×) were randomly selected from each tissue sample. According to the Fromowitz semiquantitative grading method for positive cells, the staining results were evaluated based on positive staining and the number of positive cells to create a score (26). The percentage, intensity and intracellular distribution of the staining in tumor cells (TCs) and immune cells (ICs) were evaluated separately by two pathologists. The intensity of the immunostaining of the tumor samples was graded as negative, weak, moderate or strong.

To obtain multispectral images, the stained slides were scanned using the Mantra System (PerkinElmer), which captures fluorescent spectra at 20-nm wavelength intervals from 420 nm to 720 nm with identical exposure times; the scans were combined to build a single-stack image. Images of unstained and single-stained sections were used to extract the spectrum of autofluorescence of the tissue samples and each fluorophore, respectively. The extracted images were further used to establish the spectral library required for multispectral unmixing by using InForm image analysis software (PerkinElmer). Using this spectral library, we obtained reconstructed images of the sections with the autofluorescence removed.

### Statistical Methods

The p-values and R2 values of the data were analyzed using SPSS 22.0 statistical analysis software (IBM Corporation, New York, USA). The p-values were analyzed by the chi-square test or Fisher’s test, and the R2 values were analyzed by the Pearson correlation coefficient. tumor-specific survival was calculated using the Kaplan-Meier method. A p-value < 0.05 indicated a significant difference.

## Results

### Single-Cell RNA-Seq-Based Identification of Breast Cancer-Associated Immune Cell Populations

**Figure 1:**
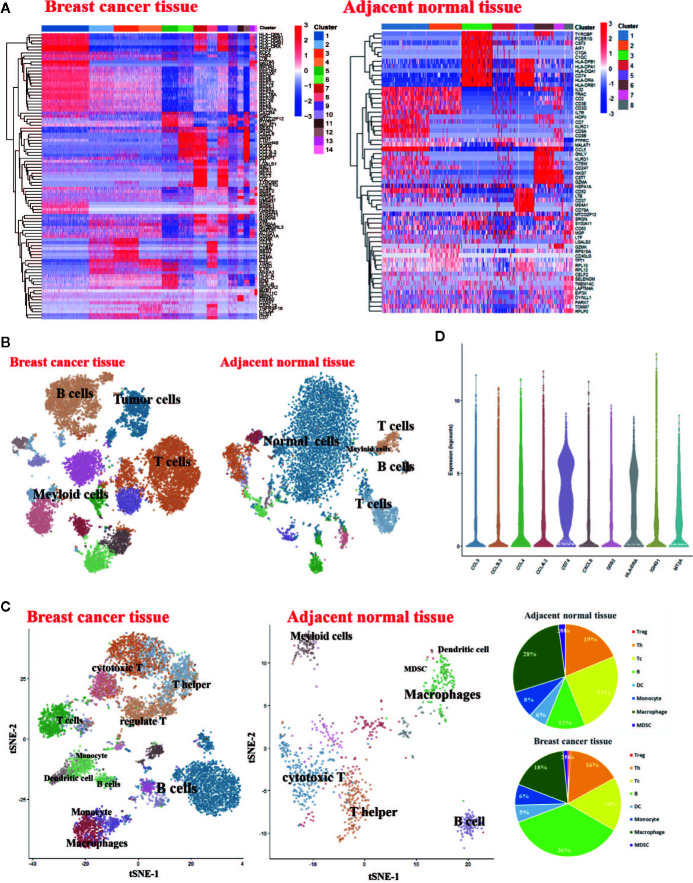
Single-Cell RNA-Seq Experimental Initial Data Exploration **(A)** Heatmap of log counts of genes in signature. **(B)** t-SNE of complete cells isolated from the breast cancer tissue and matched adjacent normal breast tissue. **(C)** t-SNE of CD45+ cells isolated from the breast cancer tissue and matched normal breast tissue (Left). Pie charts of cell-type fractions for the patient’s tumor-infiltrating immune cells, colored by cell type (Right). **(D)** The violin plot shows the top 10 most variable genes among different cells in the breast cancer sample.

To generate a transcriptional map of human breast cancer, we constructed an atlas comprising of 14897 cells including 7534 CD45+ cells collected from one primary breast carcinomas patient. To assess the effect of the tumor microenvironment on immune cell phenotypes, we also analyzed 7,320 cells from matched adjacent normal breast tissue from fresh surgical specimens. The corresponding cell populations were subjected to single-cell RNA sequencing (scRNA-seq) using 10 × platforms techniques. We first verified the major cell types in patient using PhenoGraph Clustering. We analyzed the gene expression differences between each single cluster and all other cells to identify the cluster marker genes. There are fourteen cell clusters in breast cancer tissue, while eight clusters in adjacent normal tissue (**Figure 1A**). Subsequently, we used t-distributed stochastic neighbosr embedding (tSNE) visualization of the cells to reveal major clusters in breast cancer tissue and adjacent normal tissue. An overview of cell distribution is shown in **Figure 1B**. Leukocytes are distinguished by the expression of CD45. Cell-specific markers were used to further identify the distribution of most expected immune cell types, including T cells, B cells, monocytes, macrophages and dendritic cells. There are more immune cells in breast cancer tissue than adjacent normal tissue (**Figure 1C**). The violin plot shows upregulated molecules, such as CD74, HLA-DRA, and CCL4 among others. CD74/HLA-DRA is receptor/ligand expressed in Macrophage/Monocyte functioning as MHC class II antigen processing. CCL4 and CCL4L2 are secreted proteins and have chemokinetic and inflammatory functions. The result of violin plot reveals that very active immune response existing in the breast cancer microenvironment (**Figure 1D**).

### Immune Checkpoints Expressions Heterogeneity in Immune Cell Populations in the Breast Cancer Tissue

**Figure 2:**
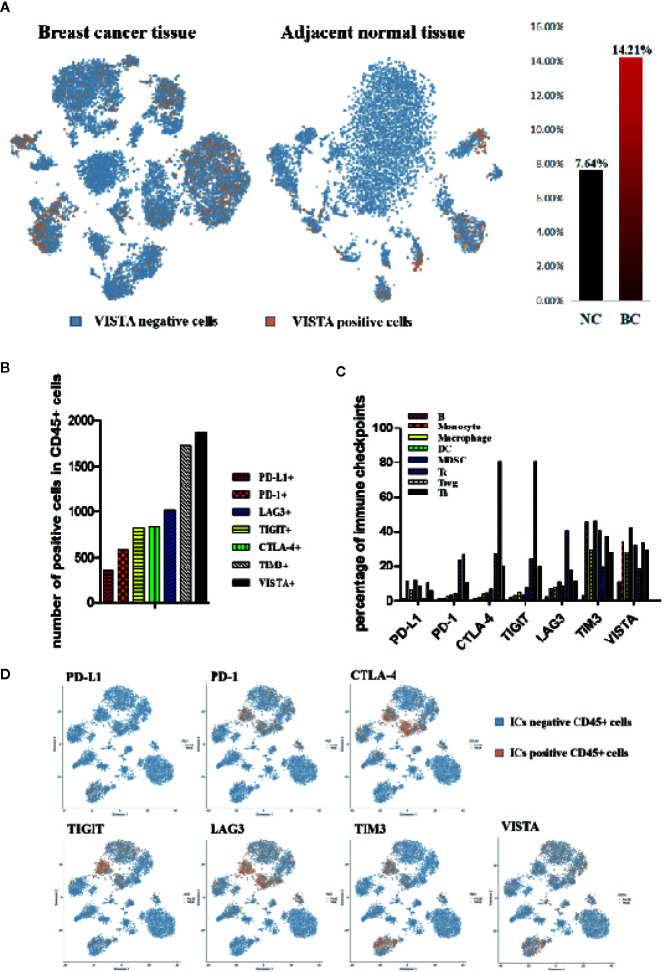
Immune Checkpoints expressions heterogeneity in immune cell populations in the breast cancer tissue. **(A)** t-SNE of normalized single-cell RNA-seq data for VISTA colored by markers. VISTA positive cells (orange), VISTA negative (blue). **(B)** Histogram of the expression of each immune checkpoint (PD-L1, PD-1, CTLA-4, TIGIT, LAG3, TIM3 and VISTA) in the microenvironment of breast cancer tissue. **(C)** Histogram of percentage of each immune checkpoint (PD-L1, PD-1, CTLA-4, TIGIT, LAG3, TIM3 and VISTA) in the primary immune cell subpopulation in the breast cancer microenvironment. **(D)** t-SNE of normalized single-cell RNA-seq data for ICs (immune checkpoints) colored by markers. ICs positive cells (orange), ICs negative (blue).

The results of sc-RNA sequence analysis showed that the expression of VISTA in breast cancer tissues (BC, 14.21%) was significantly higher than that of adjacent normal tissues (NC, 7.64%) and there was significant VISTA expression in the cancer cell area (**Figures 1B**, **2A**). In the microenvironment of breast cancer, the expression of each immune checkpoint is quite different. All the immune checkpoints we tested (PD-1, PD-L1, CTLA-4, TIM3, TIGIT, LAG3, and VISTA) were expressed on immune cells, and the number of VISTA^+^ cells was the highest (**Figure 1B**). PD-1, LAG3, TIGIT, and CTLA-4 were significantly expressed on T lymphocytes, while PD-L1, TIM3, and VISTA were mainly expressed on myeloid cells (**Figure 2B**). By detecting the proportion of cells with positive immune checkpoints in each immune subpopulation, we found heterogeneity in the expression of immune checkpoint proteins in the immune microenvironment of breast cancer. PD-1, LAG3, TIGIT, and CTLA-4 are significantly expressed on T lymphocytes, while PD-L1, TIM3, and VISTA are mainly expressed on myeloid cells (**Figures 1C**, **2C, D**).

### VISTA Expression Pattern in Human Breast Cancer Tissue Microarrays

To further support our findings, a total of 343 patients who fulfilled all study criteria were tested, and the tested samples included 4 normal breast tissue samples, 2 breast adenosis tissue samples, 13 paracancerous tissue samples and 324 breast cancer tissue samples. The clinicopathological characteristics of our sample cohort are summarized in Table S1 (**Additional file 2: Table S1**).

**Figure 3:**
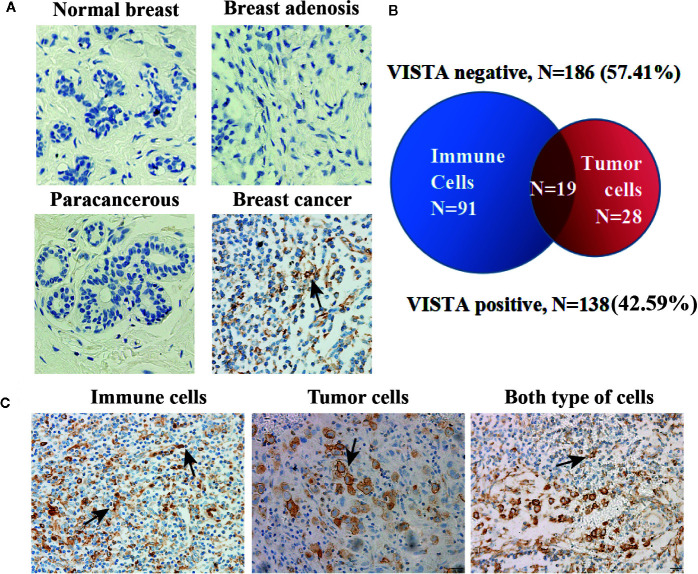
VISTA expression pattern in human breast cancer tissue. **(A)** Representative immunohistochemical staining for the VISTA protein in normal breast tissue (n=4), breast adenosis tissue (n=2), paracancerous tissue (n=13) and breast cancer tissue (n=324). Original magnification, 400 ×. **(B)** The expression of VISTA in breast cancer detected by immunohistochemistry. VISTA negative (n=186, 57.41%) vs. VISTA positive (n=138, 42.59%). **(C)** Representative positive VISTA staining in immune cells (110/324, 33.95%), tumor cells (47/324, 14.51%), and both types of cells (19/324, 5.86%). Original magnification, 200 ×.

VISTA expression was observed in tumor and immune cells but not in normal breast tissue, adenosis tissue or paracancerous tissue (**Figure 3A**). A total of 138 of the 324 patients (42.59%) showed VISTA expression in their breast cancer tissue samples with a membranous/cytoplasmic staining pattern. VISTA protein was observed not only in intratumoral immune cells (33.95%) but also in breast cancer cells (14.51%) (**Figures 3B, C**).

### VISTA Expression and the Breast Cancer Immune Microenvironment

**Figure 4:**
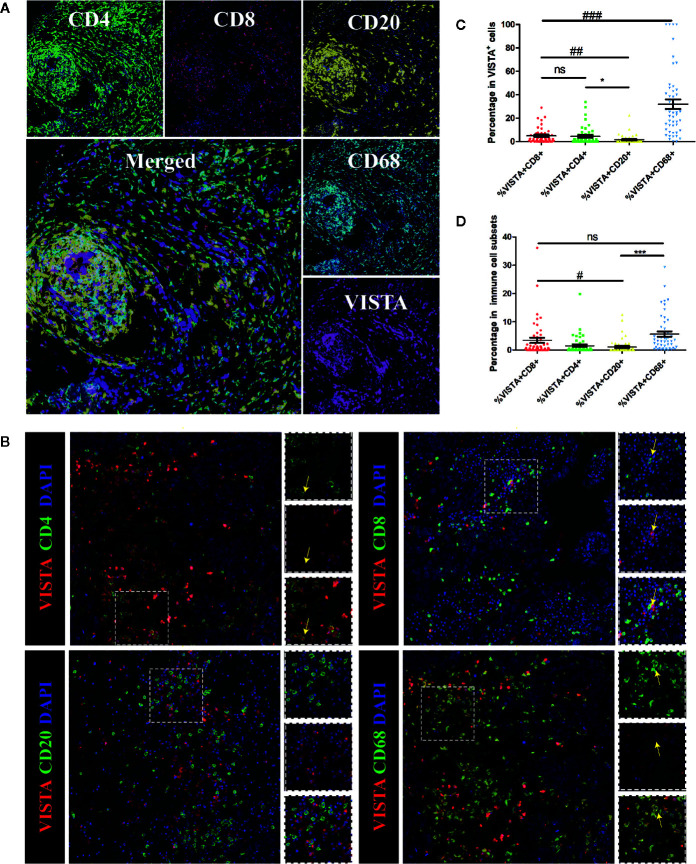
Multiplex immunofluorescence for VISTA and selected tumor-infiltrating immune cell markers in human breast cancer tissue. Markers of tumor-infiltrating immune cells: CD68 (macrophages), CD4 (T cells), CD8 (cytotoxic T cells) and CD20 (B cells). **(A)** Representative images of biomarkers in human breast cancer tissue. CD4 staining is shown in green; CD8 staining is shown in red; CD20 staining is shown in yellow; CD68 staining is shown in cyan; VISTA staining is shown in magenta; and DAPI staining is shown in blue. 400×. **(B)** Co-localization of VISTA with selected tumor-infiltrating immune cell markers in breast cancer detected by immunofluorescence. CD4, CD8, CD20, and CD68 staining is shown in green; VISTA staining is shown in red; and DAPI staining is shown in blue. Areas of co-localization are indicated with yellow arrows. 400×. **(C)**. Summary plot of the proportion of each subpopulation of cells among double-positive cells. Each dot represents data from an individual patient. P-values were obtained by an unpaired T-test. ^*^ *p* < 0.05; ^##^ *p* < 0.01; ^###^ *p* < 0.001. **(D)** Summary plot of the proportion of double-positive cells in each subpopulation of cells. Each dot represents data from an individual patient. P-values were obtained by an unpaired T-test. ^#^ *p* < 0.05; ^***^ *p* < 0.001. NS, no significance.

To further investigate the expression pattern of VISTA in breast cancer, multiplex immunofluorescence staining was used to detect the expression of VISTA in the immune cell subsets of breast cancer tissue microarray samples, which included 4 normal breast tissue samples, 2 breast adenosis tissue samples, 5 paracancerous tissue samples, and 49 breast cancer tissue samples. The results indicated that VISTA expression was detected in T cells (CD4^+^ cells and CD8^+^ cells) and macrophage cells (CD68^+^ cells) but was almost not detected in B cells (CD20^+^ cells) (**Figure 4**). Moreover, VISTA expression was higher in CD68^+^ tumor-associated macrophages (TAMs; 32.58%) than in CD4^+^ T cells (4.97%), CD8^+^ cytotoxic T cells (4.48%), or CD20^+^ B cells (1.46%) (**Figures 4C, D**).

### VISTA Expression and Patient Clinicopathological Characteristics

To explore the clinical significance of VISTA in breast cancer, we analyzed the associations between VISTA expression and patient clinicopathological characteristics. VISTA expression in breast cancer tissue was positively correlated with pathological grade (I-II compared with III; *p* = 0.001), lymph node status (comparisons among N0, N1, N2, and N3; *p* = 0.045) and genotype (comparisons among luminal, HER 2^+^ and basal-like subtypes; *p* < 0.000) (**Table 1**).

**Table 1:** Expression of VISTA in breast cancer subpopulations with different clinicopathological characteristics.

Clinicopathological characteristic	Group	Number	VISTA		*P*-value
			Pos (n, %)	Neg (n, %)	
	≥55	163 (50.77%)	76 (46.63%)	87 (53.37%)	
Age (n = 321)					0.146
	<55	158 (49.22%)	61 (38.61%)	97 (61.39%)	
Pathological Grade (n = 299)	I-II	171 (57.19%)	59 (34.50%)	112 (65.97%)	
					0.001*
	III	128 (42.81%)	69 (53.91%)	59 (46.097%)	
	T1	95 (31.46%)	38 (40.00%)	57 (60.00%)	
T-category (n = 302)	T2	188 (62.25%)	80 (42.55%)	108 (57.45%)	0.354
	T3-T4	19 (6.29%)	11 (57.89%)	8 (42.11%)	
	N0	189 (81.81%)	89 (47.09%)	100 (52.91%)	
Lymph Node Status (n = 231)	N1	51 (22.08%)	15 (29.41%)	36 (70.59%)	
					0.045*
	N2	48 (20.78%)	18 (37.50%)	30(62.508%)	
	N3	7 (3.03%)	5 (71.43%)	2(28.57%)	
	Luminal	94 (48.45%)	30 (31.91%)	64(68.09%)	
Genotype (n = 194)	HER 2^+^	7 (3.61%)	3 (42.86%)	4(57.14%)	<0.001*
	Basal-like	93 (47.94%)	64 (68.82%)	29(31.18%)	

VISTA, V-domain Ig suppressor of T cell activation; Pos, positive; Neg, negative.

In bold: *p < 0.05, Indicates a statistically significant difference.

### VISTA Expression in Breast Cancer Tissue With PD-1, PD-L1, TIGIT, TIM3, or LAG3 Expression

**Figure 5:**
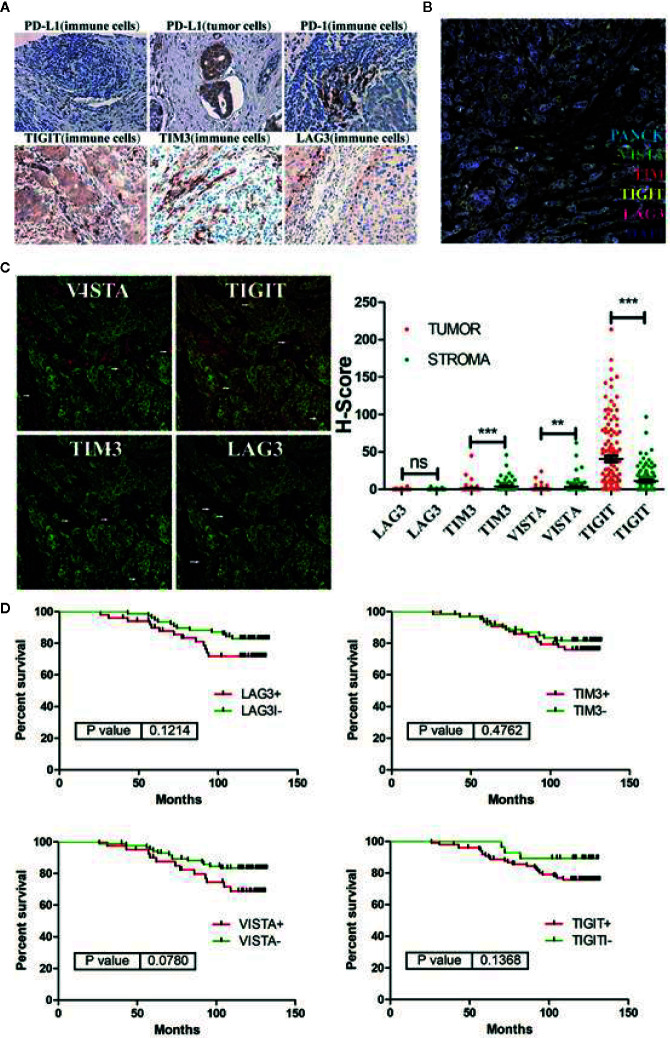
**(A)** Representative immunohistochemical staining for immune checkpoint molecules (PD-L1, PD-1, TIGIT, TIM3 and LAG3) in breast cancer samples. **(B)** Representative multiplex immunofluorescence staining for immune checkpoint molecules (VISTA, TIGIT, TIM3 and LAG3) in breast cancer samples. VISTA staining is shown in green; TIGIT staining is shown in yellow; TIM3 staining is shown in red; LAG3 staining is shown in magenta; PANCK staining is shown in cyan; and DAPI staining is shown in blue. **(C)** Co-localization of PANCK with VISTA, TIGIT, TIM3 and LAG3 in breast cancer detected by immunofluorescence. VISTA, TIGIT, TIM3, LAG3 staining is shown in red; PANCK staining is shown in green; and DAPI staining is shown in blue. **(D)** Kaplan-Meier curves showing overall survival (OS) of breast cancer patients based on immune-checkpoints (VISTA, TIGIT, TIM3 and LAG3) status. **p < 0.01, ***p < 0.001. NS, no significance.

As shown in previous reports, VISTA and PD-1 non-redundantly regulate murine T cell responses. In this study, the expression patterns of immune checkpoints, such as PD-1, CTLA-4, TIGIT, and LAG3, are quite different from that of VISTA in breast cancer microenvironment (**Figure 2**). To determine whether these combined predictors of cancer prognosis were applicable in breast cancer, we additionally examined the expression of PD-1, PD-L1, TIGIT, TIM3, and LAG3 in breast cancer tissue microarrays. The results showed that the expression of VISTA was significantly correlated with the expression of PD-1 (PD-1^+^ compared with PD-1^-^; p = 0.038) and was not associated with the expression of PD-L1, TIGIT, TIM3, LAG3 (**Table 2** and **Figure 5**).

**Table 2:** Correlation analysis between expression of VISTA protein and expression of other immune checkpoints in breast cancer patients.

Clinicopathological characteristic	Group	Number	VISTA		*P*-value
			Pos (n, %)	Neg (n, %)	
	Pos	38 (30.89%)	17 (44.74%)	21 (55.26%)	
PD-1 (n = 123)					0.038*
	Neg	85 (69.11%)	22 (25.88%)	63 (74.12%)	
	Pos	31 (23.31%)	4 (12.90%)	27 (87.10%)	
PD-L1 (n = 133)					0.204
	Neg	102 (76.69%)	24 (23.53%)	78 (76.47%)	
	Pos	119 (93.70%)	39 (32.77%)	80 (67.23%)	
TIGIT (n = 127)					N/A
	Neg	8 (6.30%)	0 (0.00%)	8 (100.00%)	
	Pos	75 (59.06%)	32 (42.67%)	43 (57.33%)	
TIM3 (n = 127)					0.052
	Neg	52 (40.94%)	7 (13.46%)	45 (86.54%)	
	Pos	7 (5.51%)	2 (28.57%)	5 (71.43%)	
LAG3 (n = 127)					0.900
	Neg	120 (94.49%)	37 (30.83%)	83 (69.17%)	

VISTA, V-domain Ig suppressor of T cell activation; Pos, positive; Neg, negative.

In bold: *p < 0.05, indicates a statistically significant difference.

In addition, in 128 breast cancer patients with a defined survival period data, the Kaplan-Meier results showed that immune checkpoints, such as VISTA (VISTA^+^ compared with VISTA^-^; *p* = 0.078), TIGIT (TIGIT^+^ compared with TIGIT^-^; *p* = 0.137), TIM3(TIM3^+^ compared with TIM3^-^; *p* = 0.176), and LAG3 (LAG3^+^ compared with LAG3^-^; *p* = 0.121), were not associated with overall survival.

## Discussion

Despite major advances in cancer immunotherapy, our ability to understand mechanisms of action or predict efficacy is confounded by the heterogeneous composition of immune cells within tumors, especially the checkpoints expression in the immune cells. The precise identity of immune-checkpoints is poorly characterized in breast cancer. Here, we have applied single-cell RNA-seq as an unbiased profiling strategy to interrogate and classify the immune checkpoints expressions in immune cells in breast cancer (**Figures 1** and **2**).

This study evaluated VISTA expression in a large cohort of human breast cancer patients. Recently, a newly published study also used immunohistochemistry to study the expression of VISTA in 919 cases of ductal carcinoma, and found that the expression of VISTA on immune cells was associated with a favorable prognosis (27). Consistent with the study, our data showed that VISTA was expressed in a substantial number of immune cells. Additionally, breast tumor cells themselves also expressed VISTA with a distinct membranous/cytoplasmic VISTA expression pattern, exacerbating the immunosuppressive milieu within the tumor microenvironment (TME) (**Figure 3**). In contrast, in this study, VISTA is significantly related to the poor prognosis factor of cancer based on the expression of VISTA in the whole organization. What is the relationship between VISTA expression and cancer prognosis needs further study.

A high level of an immune checkpoint molecule expressed in immune cells might induce immune escape. Consistent with previous findings, our findings showed a high expression level of the immune checkpoint molecule VISTA in the TME, and the protein expression of VISTA showed strong individual differences, as was the case with other immune checkpoint molecules. Franz Villarroel-Espindola et al. found that in non-small cell lung cancer, VISTA expression was significantly higher in T cells than in CD68^+^ macrophages and that higher levels of VISTA were found in CD8^+^ cytotoxic cells than in CD4^+^ T lymphocytes (15). In contrast to that study, this study found that VISTA was mainly expressed in CD68^+^ macrophages (mean = 32.58%) in breast cancer and in CD4^+^ T cells (mean = 4.97%), and lower levels of VISTA were found in CD8^+^ cytotoxic cells (mean = 4.48%) (**Figure 4**).

Immunotherapy targeting the first generation of immune checkpoint molecules (CTLA-4 and PD-1) has been proven to be effective in many cancers. However, not all patients respond to immune checkpoint inhibitors, which lead to a reduced objective response rate in the overall patient population and limits the progress of immunotherapy (9). Therefore, combination therapy with multiple immune checkpoint inhibitors involving different pathways, such as the combination of OPDIVO (nivolumab, an anti-PD-1 antibody) and Yervoy (ipilimumab, an anti-CTLA-4 antibody), has emerged and has been proven to have improved antitumor efficacy. In this study, immune checkpoint proteins were highly expressed in breast cancer patients and the single immune checkpoint (TIM3, LAG3, VISTA and TIGIT) expression is not significantly associated with breast cancer survival (**Figure 5**). This also seems to explain the poor efficacy of anti-immunization checkpoint drugs used as monotherapies in the treatment of breast cancer. In addition, VISTA expression is not associated with the expression of PD-L1, TIGIT, TIM3, and LAG3 in breast cancer, indicating that multiple immune checkpoint proteins cooperate to inhibit anti-tumor immunity and that the combined targeting of these molecules may be synergistic.

TAMs can help tumor cells proliferate and migrate and aid tumor progression. In addition, recent studies have shown that macrophages can disable immune checkpoint therapy by endocytosing anti-PD-L1 antibodies (28). Our data indicated that VISTA was specifically expressed on TAMs in breast cancer. These TAM-dependent effects result in a stimulatory TME. It is reasonable to suspect that the reprogramming of TAMs following VISTA blockade promotes T cell infiltration and activation. Further experimental proofs are ongoing.

## Conclusions

In summary, this is the first evaluation of the expression pattern of VISTA in the tumor microenvironment of breast cancer patients. We accordingly show that VISTA is highly expressed on tumor-associated macrophages. The uncorrelations between the expression levels of VISTA and other immune checkpoint molecules (PD-L1, TIGIT, TIM3, and LAG3) show the possibility of a multi-immune escape mechanism.

## Data Availability Statement

The datasets presented in this study can be found in online repositories. The names of the repository/repositories and accession number(s) can be found below: https://www.ncbi.nlm.nih.gov/, SPR234770.

## Ethics Statement

The study was conducted in accordance with the Declaration of Helsinki, and the protocol was approved by the Medical Ethics Committee of the Shanghai Outdo Biotech Company and performed according to institutional guidelines (No. YB M-05-02). The patients/participants provided their written informed consent to participate in this study.

## Author Contributions

Study concept and design: LW and JL. Experiments: XX and XH. sc-RNA sequence data analysis and interpretation of data: ZS, YW, XX, WL, and JJ. Intellectual input: JJ, CQ, and WC. Drafting the manuscript: JL and XX. Critical review of the manuscript: XX, ZS, YW, XH, CQ, LW, JJ, and JL. All authors contributed to the article and approved the submitted version.

## Funding

This research was funded by the National Natural Science Foundation of China (no. 81673443 and no. 81973361) and Double First-Class University Plan (no. CPU2018GY01). Dr. Li Wang is supported by research funding from NCI 2R01 CA164225, NCI 1R01CA223804, the Office of the Assistant Secretary of Defense for Health Affairs through the Peer Reviewed Cancer Research Program under Award No. W81XWH-14-1-0587, the Worldwide Cancer Research Foundation (UK) research grant 16-1161, and the Research Scholar Grant RSG-18-045-01 - LIB from the American Cancer Society (ACS).

## Conflict of Interest

The authors declare that the research was conducted in the absence of any commercial or financial relationships that could be construed as a potential conflict of interest.

## References

[1] Chen W Zheng R Baade PD Zhang S Zeng H Bray F Cancer statistics in China, 2015. CA Cancer J Clin (2016) 66(2):115–32. 10.3322/caac.21338 26808342

[2] PDQ® Adult Treatment Editorial Board PDQ Breast Cancer Treatment. Bethesda, MD: National Cancer Institute. Available at: https://www.cancer.gov/types/breast/patient/breast-treatment-pdq (Accessed <09/06/2019>).

[3] Flemming A Cancer: PD-1 makes waves in anticancer immunotherapy. Nature reviews. Drug Discov (2012) 11(8):601. 10.1038/nrd3806 22850780

[4] Romero D Immunotherapy: PD-1 says goodbye, TIM-3 says hello. Nature Reviews. Clin Oncol (2016) 13(4):202–3. 10.1038/nrclinonc.2016.40 26977783

[5] Postow MA Callahan MK Wolchok JD Immune Checkpoint Blockade in Cancer Therapy. J Clin Oncol (2015) 33(17):1974–82. 10.1200/JCO.2014.59.4358 PMC4980573 25605845

[6] Heppt MV Steeb T Schlager JG Rosumeck S Dressler C Ruzicka T Immune checkpoint blockade for unresectable or metastatic uveal melanoma: A systematic review. Cancer Treat Rev (2017) 60:44–52. 10.1016/j.ctrv.2017.08.009 28881222

[7] Kim DW Trinh VA Hwu WJ Ipilimumab in the treatment of advanced melanoma - a clinical update. Expert Opin Biol Ther (2014) 14(11):1709–18. 10.1517/14712598.2014.963053 25250971

[8] Khoja L Butler MO Kang SP Ebbinghaus S Joshua AM Pembrolizumab. J Immunother Cancer (2015) 18 3:36. 10.1186/s40425-015-0078-9 PMC4539882 26288737

[9] Garon EB Rizvi NA Hui R Leighl N Balmanoukian AS Eder JP Pembrolizumab for the treatment of non-small-cell lung cancer. N Engl J Med (2015) 372(21):2018–28. 10.1056/NEJMoa1501824 25891174

[10] Wang L Rubinstein R Lines JL Wasiuk A Ahonen C Guo Y VISTA, a novel mouse Ig superfamily ligand that negatively regulates T cell responses. J Exp Med (2011) 208(3):577–92. 10.1084/jem.20100619 PMC3058578 21383057

[11] Lines JL Sempere LF Broughton T Wang L Noelle R VISTA is a novel broad-spectrum negative checkpoint regulator for cancer immunotherapy. Cancer Immunol Res (2014) 2(6):510–7. 10.1158/2326-6066.CIR-14-0072 PMC4085258 24894088

[12] Liu J Yuan Y Chen W Putra J Suriawinata AA Schenk AD Immune-checkpoint proteins VISTA and PD-1 nonredundantly regulate murine T-cell responses. Proc Natl Acad Sci USA (2015) 112(21):6682–7. 10.1073/pnas.1420370112 PMC4450438 25964334

[13] Le Mercier I Chen W Lines JL Day M Li J Sergent P VISTA Regulates the Development of Protective Antitumor Immunity. Cancer Res (2014) 74(7):1933–44. 10.1158/0008-5472.CAN-13-1506 PMC4116689 24691994

[14] Blando J Sharma A Higa MG Zhao H Vence L Yadav SS Comparison of immune infiltrates in melanoma and pancreatic cancer highlights VISTA as a potential target in pancreatic cancer. Proc Natl Acad Sci USA (2019) 116(5):1692–7. 10.1073/pnas.1811067116 PMC6358697 30635425

[15] Wang L Jia B Claxton DF Ehmann WC Rybka WB Mineishi S VISTA is highly expressed on MDSCs and mediates an inhibition of T cell response in patients with AML. Oncoimmunology (2018) 7(9):e1469594. 10.1080/2162402X.2018.1469594 30228937 PMC6140587

[16] Villarroel-Espindola F Yu X Datar I Mani N Sanmamed M Velcheti V Spatially Resolved and Quantitative Analysis of VISTA/PD-1H as a Novel Immunotherapy Target in Human Non-Small Cell Lung Cancer. Clin Cancer Res (2018) 24(7):1562–73. 10.1158/1078-0432.CCR-17-2542 PMC5884702 29203588

[17] Liu J Xie X Xuan C Li T Wang L Teng L High-Density Infiltration of V-domain Immunoglobulin Suppressor of T-cell Activation Up-regulated Immune Cells in Human Pancreatic Cancer. Pancreas (2018) 47(6):725–31. 10.1097/MPA.0000000000001059 29771768

[18] Böger C Behrens HM Krüger S Röcken C The novel negative checkpoint regulator VISTA is expressed in gastric carcinoma and associated with PD-L1/PD-1: A future perspective for a combined gastric cancer therapy? Oncoimmunology (2017) 6(4):e1293215. 10.1080/2162402X.2017.1293215 28507801 PMC5414883

[19] Wu L Deng WW Huang CF Bu LL Yu GT Mao L Expression of VISTA correlated with immunosuppression and synergized with CD8 to predict survival in human oral squamous cell carcinoma. Cancer Immunol Immunother (2017) 66(5):627–36. 10.1007/s00262-017-1968-0 PMC11028774 28236118

[20] Zielinski C Knapp S Mascaux C Hirsch F Rationale for targeting the immune system through checkpoint molecule blockade in the treatment of non-small-cell lung cancer. Ann Oncol (2013) 24(5):1170–9. 10.1093/annonc/mds647 PMC3629900 23393121

[21] Liao H Zhu H Liu S Wang H Expression of V-domain immunoglobulin suppressor of T cell activation is associated with the advanced stage and presence of lymph node metastasis in ovarian cancer. Oncol Lett (2018) 16(3):3465–72. 10.3892/ol.2018.9059 PMC6096210 30127950

[22] Xie S Huang J Qiao Q Zang W Hong S Tan H Expression of the inhibitory B7 family molecule VISTA in human colorectal carcinoma tumors. Cancer Immunol Immunother (2018) 67(11):1685–94. 10.1007/s00262-018-2227-8 PMC11028359 30128738

[23] Wolchok JD Hodi FS Weber JS Allison JP Urba WJ Robert C Development of ipilimumab: a novel immunotherapeutic approach for the treatment of advanced melanoma. Ann N Y Acad Sci (2013) 1291:1–13. 10.1111/nyas.12180 23772560 PMC3910157

[24] Gao J Ward JF Pettaway CA Shi LZ Subudhi SK Vence LM VISTA is an inhibitory immune checkpoint that is increased after ipilimumab therapy in patients with prostate cancer. Nat Med (2017) 23(5):551–5. 10.1038/nm.4308 PMC5466900 28346412

[25] Schmid P Adams S Rugo HS Schneeweiss A Barrios CH Iwata H IMpassion130 Trial Investigators. Atezolizumab and Nab-Paclitaxel in Advanced Triple-Negative Breast Cancer. N Engl J Med (2018) 379(22):2108–21. 10.1056/NEJMoa1809615 30345906

[26] Fromowitz FB Viola MV Chao S Oravez S Mishriki Y Finkel G ras p21 expression in the progression of breast cancer. Hum Pathol (1987) 18(12):1268–75. 10.1016/s0046-8177(87)80412-4 3315956

[27] Zong L Mo S Yu S Zhou Y Zhang M Chen J Expression of the immune checkpoint VISTA in breast cancer. Cancer Immunol Immunother (2020) 69(8):1437–46. 10.1007/s00262-020-02554-3 PMC11027729 32266446

[28] Arlauckas SP Garris CS Kohler RH Kitaoka M Cuccarese MF Yang KS In vivo imaging reveals a tumor-associated macrophage-mediated resistance pathway in anti-PD-1 therapy. Sci Transl Med (2017) 9(389):eaal3604. 10.1126/scitranslmed.aal3604 28490665 PMC5734617

